# Genomic Characterization of a Novel Virus of the Family *Tymoviridae* Isolated from Mosquitoes

**DOI:** 10.1371/journal.pone.0039845

**Published:** 2012-07-27

**Authors:** Lihua Wang, Xinjun Lv, Yougang Zhai, Shihong Fu, David Wang, Simon Rayner, Qing Tang, Guodong Liang

**Affiliations:** 1 State Key Laboratory for Infectious Diseases Prevention and Control, Institute for Viral Disease Control and Prevention, Chinese Center for Disease Control and Prevention, Beijing, China; 2 Washington University, St. Louis, Missouri, United States of America; 3 State Key Laboratory for Virology, Wuhan Institute of Virology, Chinese Academy of Sciences, Hubei, China; University of Kansas Medical Center, United States of America

## Abstract

**Background:**

The family *Tymoviridae* comprises three plant virus genera, including *Tymovirus*, *Marafivirus*, and *Maculavirus*, which are found in most parts of the world and cause severe agricultural losses. We describe a putatively novel member of the family *Tymoviridae*, which is isolated from mosquitoes (*Culex spp.*), referred to as CuTLV.

**Methods and Results:**

The CuTLV was isolated by cell culture, which replicates and causes cytopathic effects in *Aedes albopictus* C6/36 cells, but not in mammalian BHK-21 or Vero cells. The complete 6471 nucleotide sequence of CuTLV was determined. The genome of CuTLV is predicted to contain three open reading frames (ORFs). The largest ORF1 is 5307 nucleotides (nt) in length and encodes a putative polypeptide of 1769 amino acids (aa), which contains the conserved motifs for the methyltransferase (MTR), Tymovirus endopeptidase (PRO), helicase (HEL), and RNA-dependent RNA polymerase (RdRp) of the replication-associated proteins (RPs) of positive-stranded RNA viruses. In contrast, the ORF1 sequence does not contain the so-called “tymobox” or “marafibox”, the conserved subgenomic RNA promoter present in all tymoviruses or marafiviruses, respectively. ORF2 and ORF3 putatively encode a 248-aa coat protein (CP) and a proline-rich 149-aa polypeptide. The whole genomic nucleotide identity of CuTLV with other members of family *Tymoviridae* ranged from 46.2% (ChiYMV) to 52.4% (GFkV). Phylogenetic analysis based on the putative RP and CP genes of CuTLV demonstrated that the virus is most closely related to viruses in the genus *Maculavirus*.

**Conclusions:**

The CuTLV is a novel virus related to members of the family *Tymoviridae*, with molecular characters that are distinct from those of tymoviruses, marafiviruses, and other maculaviruses or macula-like viruses. This is the first report of the isolation of a Tymoviridae-like virus from mosquitoes. Further investigations are required to clarify the origin, replication strategy, and the public health or agricultural importance of the CuTLV.

## Introduction

There are three genera (*Tymovirus, Marafivirus, Maculavirus*) in the family *Tymoviridae* [1], [2]. Viruses in this family have the following characteristics: a positive-sense, single-stranded RNA (ssRNA) genome with an unusually high cytosine content (range, 32% to 50%); a genome length that ranges from 6.0 kb to 7.5 kb; capping at the 5′-terminus; and the presence of open reading frames (ORFs) that encode replication-related proteins analogous to those of other taxa of the “alpha-like” supergroup of ssRNA viruses [1]. The genomic organization and number of ORFs in the family *Tymoviridae* differ according to the genus or individual viral species. The genomic RNA of tymoviruses is 6.0–6.7 kb in size, contains three ORFs, has a 16-nucleotide (nt) sequence known as the “tymobox” which functions as a subgenomic RNA promoter, and has a tRNA-like 3′-terminal structure [1]–[3]. The distinctive feature of the marafivirus genome (6.0–6.5 kb) is the presence of a large single ORF that contains the “marafibox”, which is a conserved 16-nt region comparable to the tymobox, from which it differs by two or three residues and the possession a poly(A) tail at the 3′-terminus [1], [2], [4]. The genomic RNA of maculaviruses (approximately 7.5 kb) is the largest in the family, consisting of four ORFs with a polyadenylated 3′-terminus, and it lacks a conserved sequence comparable to the tymobox or marafibox [1], [2], [5]. The replication strategy of these viruses in the family *Tymoviridae* is thought to involve post-translational proteolytic cleavage of the polypeptide encoded by the large ORF by a papain-like virus-encoded protease, as well as coat protein expression *via* a subgenomic RNA [1], [6].

To date, 26, 7, and 1 known species have been reported for the genera *Tymovirus*, *Marafivirus*, and *Maculavirus,* respectively. In addition, several unclassified viruses which may be members of these genera but have not been approved as species have been reported recently [2]. Natural vectors for tymoviruses and marafiviruse*s* are coleoptera and leafhoppers, which transmit in a semi-persistent or persistent manner [1], [2]. The vectors for maculaviruses are unknown [1], [5]. In the present study, we describe molecular properties of a novel Tymoviridae-like virus isolated from mosquitoes (*Culex spp.*), referred to as CuTLV, and its phylogenetic relationships with known members of this family.

## Results

### Virus isolation

**Figure 1:**
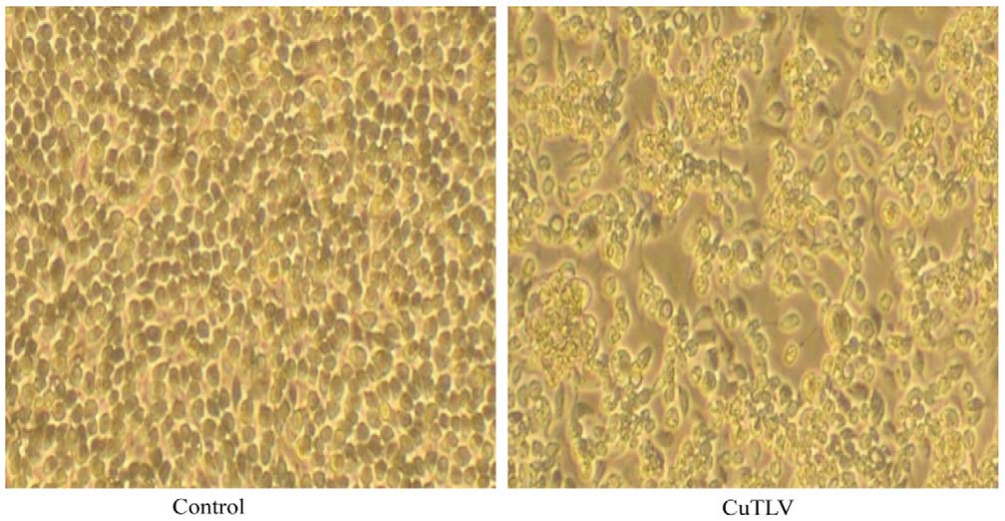
Cytopathic effects of CuTLV on C6/36 cells after three days of infection (200X).

The CuTLV virus was isolated from one pool of mosquitoes (*Culex spp.*) captured in Xinjiang, China in 2005. The virus was found to induce cytopathic effect (CPE) in first-passage C6/36 (*Aedes albopictus*) cells on Day 3 after inoculation. The CPE was characterized by cell shrinking, shedding, fusion or cytolysis with eventual detachment from the growth surface (Fig. 1). No CPE was observed in mammalian BHK-21 and Vero cells even after three rounds of blind passage (at 7 days per cycle).

### Virus identification and whole genome sequencing

The antigens from virus-inoculated C6/36 cell lysates did not react with antisera to known arboviruses of the genera *Alphavirus*, *Flavivirus*, *Bunyavirus*, and *Seadornavirus* [7]–[10] isolated in China, suggesting that this virus is an uncommon arbovirus that is carried or transmitted by mosquitoes in China.

**Figure 2:**
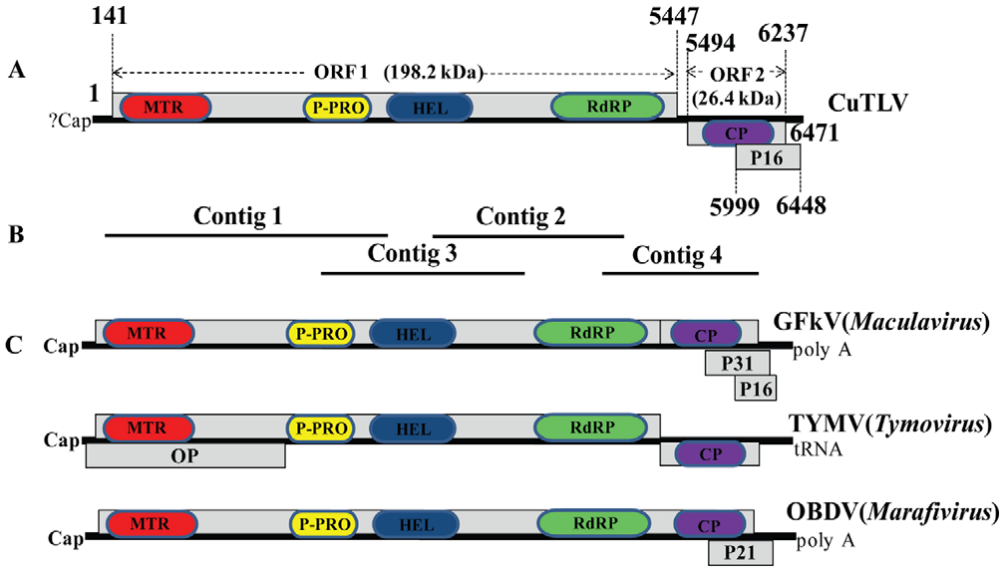
Comparison of the genomic organization of CuTLV with GFkV, TYMV and OBDV. (A) Genomic organization of CuTLV; (B) Overlapping contigs obtained by DNase-SISPA; (C) Genomic organization of GFkV, TYMV and OBDV. ORFs are indicated by grey boxes. The putative conserved domains showed in the boxes by different color (Blast was performed for Conserved Domain Database in NCBI). MTR, methyltranferase; P-PRO, papain-like protease; HEL, helicase; RdRp, RNA dependent RNA polymerase protein; CP, coat protein; OP, movement protein; p31, p21 and p16 are proline-rich proteins.

Total RNA was extracted from CuTLV inoculated C6/36 cell lysates and analyzed independently in two laboratories (Washington University School of Medicine, St. Louis, MO, USA, and State Key Laboratory for Infectious Diseases Prevention and Control, IVDR, China CDC). The extracted RNA was randomly PCR amplified and hybridized to a pan-viral DNA microarray as described [10]. Multiple probes derived from viruses in the family *Tymoviridae* gave strong hybridization intensities (data not shown). DNase-SISPA (sequence-independent single primer amplification) [11] yielded 4 unique bands that were cloned and sequenced. From these sequences, a contig of 5380 nt was assembled (Fig. 2), which had 36.7–50% amino acid sequence identity to viruses in the family *Tymoviridae* following BLAST searches.

To obtain the whole genome sequence of CuTLV, 5′ RACE and a modified 3′ RACE protocol were performed. The entire genomic sequence was confirmed by overlapping PCR amplification and sequencing with primers (Table 1) designed according the sequences obtained from the SISPA and RACE analyses. The complete genomic RNA (gRNA) of CuTLV consists of 6471 nt and has been deposited in GenBank (JQ429443).

**Table 1:** Primers for overlapping RT-PCR confirmation of CuTLV whole genome.

Primer Name	Direction	Sequence (5′-3′)	Position	Products(bp)
F01	Sense	GCATTTAAACAAAACAACACCCAG	1–24	821
R01	Antisense	TTGGGCGGCTGAGAGAGTG	803–821	
F02	Sense	TATCGGTACTACTCCGCTAAAGG	711–733	862
R02	Antisense	AAGTAGTAGAGGACCGTGACAGC	1551–1573	
F03	Sense	CAATCTTCTCGCAGCCATCC	1409–1428	993
R03	Antisense	ACCGTTGGTTTGTGTTTCAGAG	2381–2402	
F04	Sense	GGCACACAGTGAATGTCACAGC	2188–2209	810
R04	Antisense	CAGGTGGAAACACGCCAAC	2980–2998	
F05	Sense	GTCTTTCTTCCGTGGTTCTGAG	2873–2894	939
R05	Antisense	GGGTAGATTCGGGATGAGGTC	3792–3812	
F06	Sense	GTGGAGTCGAACTCCTCCGTC	3625–3645	828
R06	Antisense	TGACAGGCTTTCCAGGAGGTG	4433–4453	
F07	Sense	TCTGCTCTATCAGGCTTGGTGTC	4202–4224	998
R07	Antisense	GCGGCTTGGTAGATGACTTGG	5180–5200	
F08	Sense	TTTTCTGTGGCTATCTATTGGGC	5000–5022	1005
R08	Antisense	CCAACAGATTCCATCATGTCGGTG	5981–6005	
F09	Sense	GCCAAATTCCTCCATCTCGAAGTCGT	5875–5900	596
R09	Antisense	GTTAAT TTA GTG ACG GTG TTT GG	6448–6471	

### Genome organization and characteristics of CuTLV

Like other members of family Tymoviridae, the genomic sequence of CuTLV contains a high percentage of cytosines and a low percentage of guanines (A, 21.6%; C, 39.5%; G, 14.6%; U, 24.4%) [1], [12] (Table 2). CuTLV is predicted to encode three ORFs (Fig. 2). The largest ORF (ORF1; nt 141–5447) is 5307 nt in length and encodes a putative polypeptide of 1769 aa with a predicted molecular mass of 198.2 kDa. ORF1 is a putative replication-associated polyprotein (RP), as it contains the conserved motifs of the viral RNA methyltransferase (MTR), tymovirus endopeptidase (PRO), viral RNA helicase (HEL), and RNA-dependent RNA polymerase domain (RdRP) [1], [2], [12]–[15]. In similarity to GFkV [15], ORF1 lacks the highly conserved 16-nt subgenomic RNA promoter (referred to as tymobox or marafibox) that has been identified near the end of the viral replicases of all sequenced tymoviruses [3] and marafiviruses [4].

**Table 2:** Genome and biological characters comparison of CuTLV and representatives of genera *Tymovirus, Marafivirus* and *Maculavirus.*

Family	Unassigned	Genus *Tymovirus*	Genus *Marafivirus*	Genus *Maculavirus*
Tymoviridae	CuTLV(JQ429443)	TYMV (X16378)	MRFV(AF265566)	GFkV (NC_003347)
Genome length (nt)	6 471	6 318	6 305	7 564
Nucleotide distribution	39.5%C, 14.6%G, 21.6%A, 24.4%U	39.1%C, 17.0%G, 23.0%A, 20.8%U	38.4%C, 23.6%G, 15.3%A, 22.7%U	49.9%C, 16.3%G, 13.9%A, 19.8%U
5′UTR (nt)	140, Capped(?)	88, Capped	96, Capped	291, Capped
3′UTR (nt)	23, Unpolyadenylated	109, tRNA-like	125, Polyadenylated	35, Polyadenylated
ORF number	3	3	2	4
Tymobox/marafibox	None	Have	Have	None
Host range	?	Dicotyledonous plants (Cruciferae)	Poaceae (Zea mays)	Dicotyledonous plants (European and American Vitis)
Vector	Mosquitoes	Coleopteran insects (beetles)	Hemipteran insects	Unknown
Symptoms	?	Diffuse chlorotic local lesions or systemic yellow mosaic	Maize bushy stunt	Localized clearing flecks, leaves wrinkled, twisted and varying degrees of stunting
Distribution	China	Eurasian region (in the north west), Australia, America	Argentina, Brazil, Colombia, Costa Rica, Mexico, Peru, Venezuela, Uruguay, Panama, Guatemala, Honduras, Nicaragua, Iranian and U.S.A	Europe, U.S.A and South Africa

The second ORF (ORF2; nt 5494–6237) is 747 nt in length and encodes a putative 248-aa protein with a predicted molecular mass of 26.4 kDa. ORF2 is located at the 3′-end of the CuTLV genome. The putative CP of CuTLV has low-level sequence identity with the CPs of other members of family *Tymoviridae*, ranging from 27.0% identity with *Ononis yellow mosaic virus* (OYMV, NC_001513) to 37.9% identity with *Bombyx mori macula-like virus* (BmMLV, AB186123).

ORF3 overlaps the CP gene towards the 3′-terminus. ORF 3 starts at nt 5999 and ends with an opal stop codon (UGA) at nt 6446, which is 447 nt in length and encodes a putatively proline and serine rich 149-aa polypeptide (16 kDa). The putative p16 of CuTLV shares limited homology with the proline-rich p31 (21.8%) and p16 (35.2%) proteins [14] of GFkV (NC003347).

### Characteristics of the 5′-end and 3′-end of CuTLV

**Figure 3:**
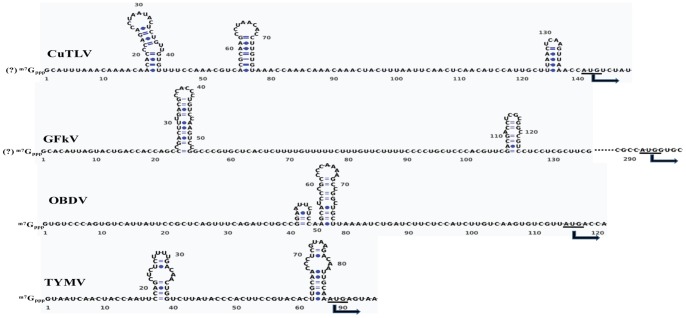
The putative secondary structure of the 5′-UTR of CuTLV RNA compared to the GFkV, OBDV and TYMV. Secondary structure of TYMV has been determined experimentally [32]. The start codons (AUG) of the RP ORFs are indicated (arrows).

The sequence of the 5′-end of the CuTLV genome was determined by 5′-RACE. The 5′-UTR of the CuTLV genome begins with a G residue, is 140 nt long, and can be folded into three stem-loops situated just upstream of the RP AUG initial codon. However, the number and location of the hairpins differ between CuTLV and other members of family *Tymoviridae* (Fig. 3). The 3′-terminal sequence of CuTLV was determined using a modified 3′-RACE method. The lack of a poly(A) tail was confirmed by the inability of the genomic RNA to act as a template for cDNA synthesis using reverse transcriptase and an oligo(dT) primer, and failure amplification for an attempted PCR using oligo(dT) and primer F09. The 3′-UTR of CuTLV RNA is 23 nt in length, which is shorter than that of other members of family *Tymoviridae* [1], [5], [12]–[15]. With a -AAC 3′-terminus, CuTLV RNA lacks the -CC(A) terminus that has to date been a property of all tymoviral RNAs [16], [17]. Although the 3′-UTR of CuTLV can be folded into a stem-loop structure, it lacks the tRNA-like secondary structure (data not shown) possessed by most tymoviruses [1], [2], [18].

### Phylogenetic classification of CuTLV

The complete nucleotide sequence of CuTLV was aligned with 21 full-length genomic sequences of the family Tymoviridae (obtained from GenBank). The overall nucleotide identities of CuTLV with other members of family Tymoviridae ranged from 46.2% to 52.4%; the highest identity (52.4%) was with GFKV (NC_003347) and the lowest identity (46.2%) was with ChiYMV (NC_014127).

**Figure 4:**
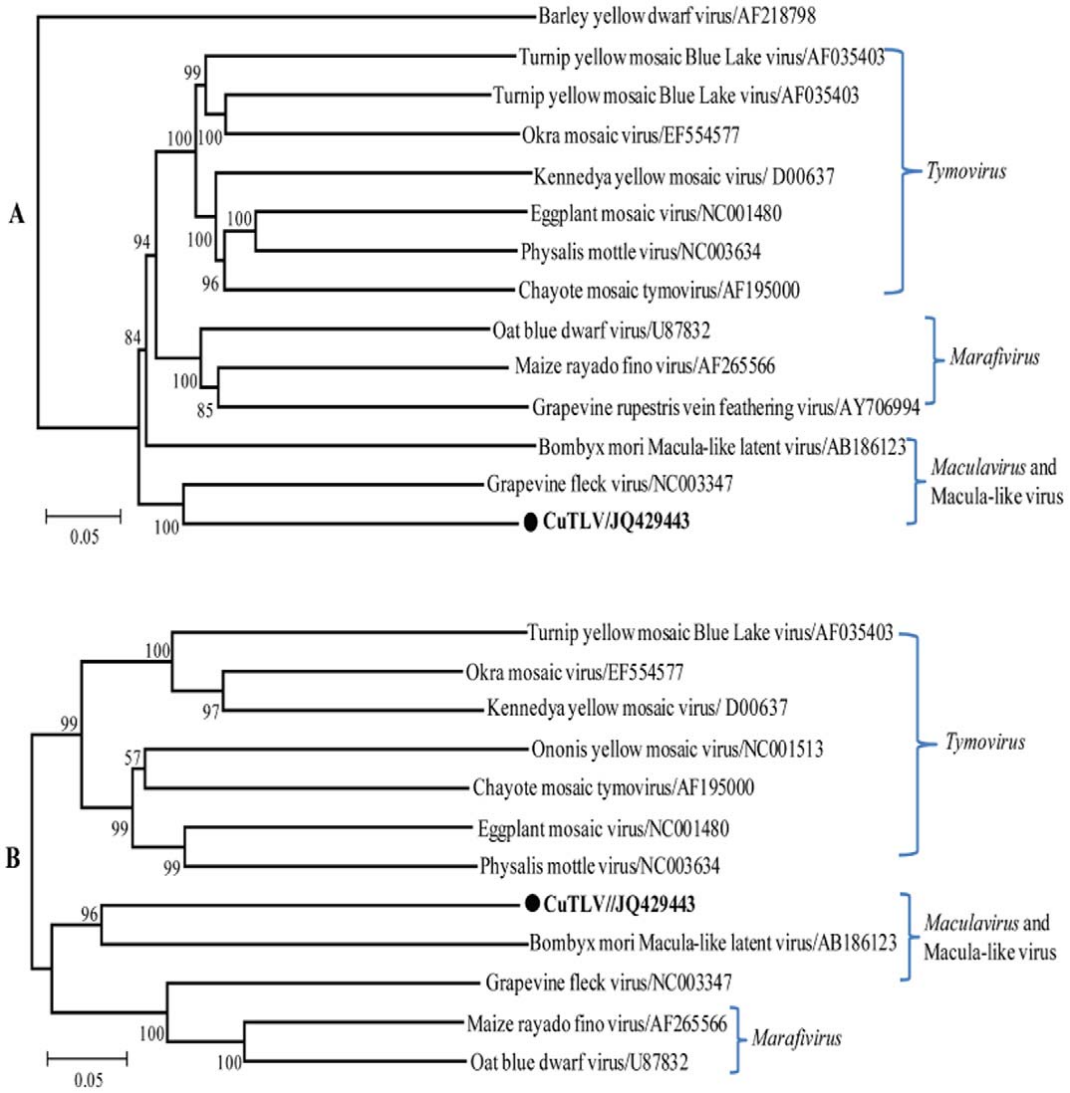
Phylogenetic tree of CuTLV based on the RP (A) and CP (B) sequences. Phylogenetic analyses were performed by the neighbor-joining method using MEGA version 4 (www.megasoftware.net). Bootstrap probabilities of each node were calculated with 1000 replicates. The RP (A) tree was rooted by using barley yellow dwarf virus sequence as the out group virus. For CP (B) tree, no outgroup was included. Horizontal branch lengths are proportional to genetic distance and vertical branch lengths have no significance. The scale indicates the number of nucleotide substitutions per site. Sequence of CuTLV from this study is in **boldface**.

**Figure 5:**
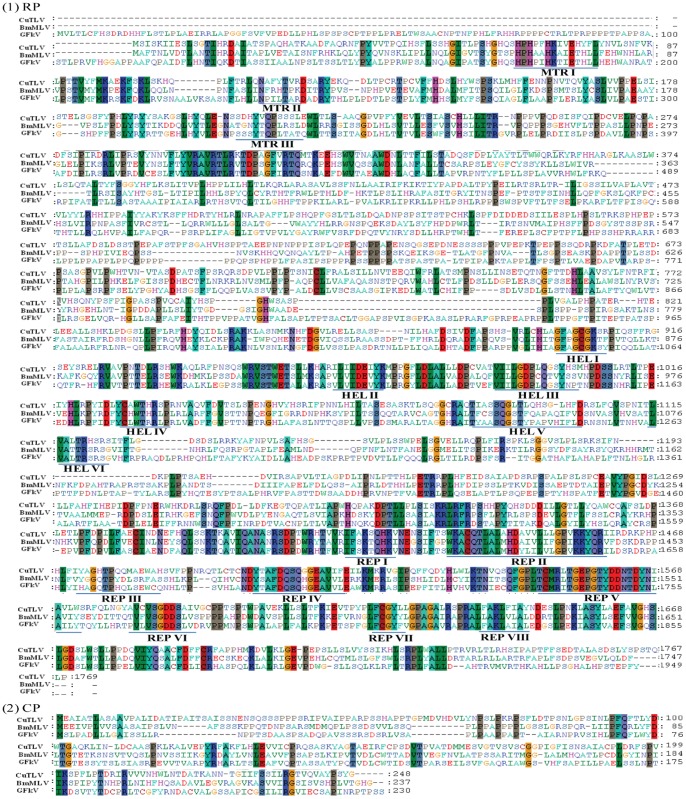
RP (1) and CPs (2) amino acid sequence alignment of CuTLV, BmMLV and GFkV. Underlining indicates conserved motifs of methyltransferase (MTR I to III), helicase (HEL I to VI), and polymerase (REP I to VIII), which were inferred from comparison with the sequence of maize rayado fino virus [14].

Phylogenetic analyses of CuTLV and other members of the *Tymoviridae* family based on the sequences of the RP and CP genes revealed that the topology of both trees was shown to coincide well. CuTLV is related to members of the *Tymoviridae* family, especially those of the *Maculavirus* including BmMLV (Bombyx mori macula-like latent virus) [19] and GFkV (the type species of *Maculavirus*) [15] (Fig. 4), the two completely sequenced maculaviruses. Alignment of amino acid sequences of RPs and CPs of CuTLV, BmMLV and GFkV revealed that the motifs (MTR I to III, HEL I to VI, and REP I to VIII) are well conserved among these viruses (Fig. 5). The amino acid identities of RdRp domain of the RPs were 60.0% and 61.4%, respectively, compared CuTLV with BmMLV and GFkV.

## Discussion

We identified a novel virus that shares many characteristics of the family *Tymoviridae*. Among the chief characteristics is a large ORF that encodes proteins associated with replication of positive sense RNA viruses. Phylogenetic analysis of the predicted RP and CP genes demonstrated that CuTLV falls within the genus *Maculavirus*. This classification is supported by the absence of a tymobox or marafibox, the 16 nt conserved sequences that act as subgenomic promoters in the genera *Tymovirus* and *Marafivirus*. However, both nucleic acid and amino acid homology are very low compared CuTLV with BmMLV and GFkV, the reported macula-like virus which identified in Bombyx mori cultured cells and the type species of *Maculavirus*. Other distinctive features of CuTLV is the presence of three predicted stem loops structures in the 5′ UTR, and non-polyadenylated genomic RNA with a -AAC 3′-terminus.

Members of the family *Tymoviridae* have been recorded from most parts of the world. Geographical distribution of individual species varies from restricted to widespread [1] (Table 2). *Tymoviruses* and *maculaviruses* mainly infect dicotyledonous plants (*Cruciferae*, European and American *Vitis* species) [1], [5], while *marafiviruses* preferentially infect *Poaceae* (*Zea mays*) [1]. Disease symptoms include a bright-yellow mosaic or mottling (tymoviruses), chlorotic stripes, white etched lines or dwarfing (marafiviruses) and flecking of the leaves (maculaviruses) [1], [5]. These plant diseases cause significant crop losses and economic burden each year. For example, in the case of *Okra mosaic virus* (*Tymovirus*), early infection of okra leads to yield losses of 12%–90% depending on the severity of the disease [20]. MRFV (*Marafivirus*) is of great agronomic importance, as it produces significant yield losses in maize throughout Central and South America [21]. The viruses in the genus *Marafivirus* are transmitted by hemipteran insects (plant hoppers; *Cicadellidae*) in a persistent manner [1]. The viruses in the genus *Tymovirus* are persistently or semipersistently transmitted by coleopteran insects (beetles) belonging to the families *Chrysomelidae* and *Curculionidae*, which are nonsucking insects. The vectors of *Maculavirus* are unknown [1]. Recently, a macula-like virus BmMLV was reported which is infectious to and replicable in B. mori-derived cells [19], [22]. So far no mosquitoes have been reported as vectors of plant viruses. Mosquitoes are capable of carrying and transmitting viruses that cause significant public health problems throughout the world [23]–[25]. The CuTLV reported in this study was isolated from mosquitoes, and it has cytopathic effects on cell lines of mosquito origin, which suggests that this virus may be able to replicate in mosquitoes. Since male mosquitoes feed on plant nectar and juices [26], we imagine that CuTLV may be transferred to mosquitoes from its host plant through this route, and started to replicate in the insect away from the plant because tymoviruses and marifiviruses can potentially replicate both in plants and in insects [1], [2].

In conclusion, we identified a novel Tymoviridae-like virus from mosquitoes, CuTLV, and analyzed its genomic characterization. Long-term monitoring and further investigations should be performed to clarify the origin and replication strategy of this virus, and the potential risks that this virus poses for agriculture or public health.

## Materials and Methods

### Cell cultures

*Aedes albopictus* C6/36 cell lines were grown in minimal essential medium (MEM; HyClone) with Hanks' salt solution supplemented with 10% FBS and 100 U/ml each of penicillin and streptomycin. The C6/36 cells were propagated and maintained at 28°C. BHK-21 and Vero cells were grown in MEM with a balanced salt solution supplemented with 10% FBS and 100 U/ml each of penicillin and streptomycin. The mammalian cells were maintained at 37°C in 5% CO_2_.

### Mosquito collection and virus isolation

To investigate arboviruses in China, mosquitoes were collected from July to August 2005 in Jiashi (39° 16′∼ 40° 00′ N, 76° 20′∼78° 00′ E), Xinjiang, China, using mosquito-trapping lamps (Wuhan Lucky Star Environmental Protection Tech, Hubei, China) placed in resting sites of animal corrals in the evening (No specific permits were required for the described field studies. No specific permissions were required for collection of mosquitoes from this location. Field studies did not involve endangered or protected species). The mosquitoes were sorted and pooled. Pools of mosquitoes were homogenized, then inoculated and blind passaged three times (7 days per cycle) on monolayers of C6/36, Vero, and BHK-21 cells in 24-well plates, as previously described [27]. The cells were observed daily for cytopathic effects and the culture fluids were stored at −80°C for further analysis. We attempted without success to identify the isolate using ELISA, IFA, and RT-PCR techniques that are well-established for specific arboviruses in our laboratory, particularly the alphaviruses (including *Mayaro virus*, *Sagiyama virus*, *Ross River virus*, *Chikungunya virus*, *Eastern* and *Western equine encephalitis viruses*, *Getah virus*, *Sindbis virus*, and *Semliki Forest virus*), flavivirus (*Japanese encephalitis virus*), bunyaviruses, and seadornaviruse*s* (*Banna virus* and *Liaoning virus*).

### Identification of isolate CuTLV using the DNase-SISPA (sequence-independent single primer amplification) method

DNase I (100 U; Stratagene) was added to 200 µl of culture fluids containing isolate CuTLV, and incubated for 2 h at 37°C, which was then used for RNA extraction using the QIAamp Viral RNA Mini kit (Qiagen) according to the manufacturer's protocol. Extracted RNA was converted to double-stranded cDNA with random hexamers. After completion of cDNA synthesis, the reaction mixtures (100 μl) were heat-inactivated at 72°C for 10 min, 10 U of the restriction endonuclease *Csp6* I (Fermentas) were added, and the mixture was incubated for 1 h at 37°C. Then, the DNA fragments were extracted, precipitated, dissolved in water, and ligated to Adaptor_A (5′-AGGCAACTGTGCTATCCGAGGGAG-3′) and Adaptor_B (5′-TACTCCCTCGG-3′) in a 20-μl reaction that contained 20 pmol of each adaptor and 1 U of T4 DNA ligase (New England BioLabs) in a standard ligation buffer. The reaction mixture was incubated at 4°C for 1 h, followed by 16°C for 4 h. Then, 2 μl of the ligation reaction was used as a template for PCR, using Adaptor A as the primer. The PCR products were analyzed on a 1.5% agarose gel, and then extracted using the QIAquick Gel Extraction kit (Qiagen). The gel-purified fragments were cloned into the pGEM-T Easy Vector (Promega), and introduced into chemically competent *Escherichia coli* TOP-10 cells (Invitrogen). After transformation, single colonies were cultured and sequenced using the ABI 3730xl sequencing system and Big Dye Terminator chemistry (Applied Biosystems). Three clones were sequenced for each extracted and cloned band. The obtained sequences were subjected to homology searching using the BLAST software and the NCBI database (http://www.ncbi.nlm.nih.gov/BLAST/).

### Genome sequencing

Contigs obtained from SISPA were assembled using the Lasergene SeqMan program (www.DNASTAR.com). Based on the sequences obtained from SISPA, the RACE method was used to identify the 5′-UTR and 3′-UTR of the CuTLV genome. The 5′-RACE method was employed using a commercially available kit (Invitrogen) with the Universal Amplification Primer (UAP) and CuTLV segment-specific primer R1 (Table 1), according to the manufacturer's specifications. A modified 3′-RACE was performed using the commercially available kit (Invitrogen). Briefly, the 3′-end of the viral RNA was ligated with an adapter (5′-P-GTCGATCACGCGATCGAACGGTCGC TGAG-3′) using T4 RNA ligase (Amersham). Reverse transcription was performed using the Ready-To-Go You-Prime First-Strand Beads (Amersham) with the short primer 5′-CAGCGACCGTTCGATCGC-3′. PCR was performed with primer F8 (Table 1) and the short primer under the following conditions: denaturation at 94°C for 5 min; 30 cycles of amplification (94°C for 30 s, 58°C for 30 s, 72°C for 2 min); and a final extension for 10 min at 72°C. The positive PCR products were cloned, and the plasmid inserts were sequenced. Ten pairs of primers (Table 1) were designed based on the sequences obtained from SISPA and RACE; these primers covered the entire genome of CuTLV and were used to amplify overlapping (100–200 bp) regions under the following conditions: denaturation at 94°C for 5 min and 30 cycles of amplification (94°C for 30 s, 58°C for 30 s, 72°C for 1 min). For double checking if poly(A) tail exist at the end of CuTLV, cDNA were prepared by oligo-dT, and attempted PCR (94°C for 30 s, 58°C for 30 s, 72°C for 1 min) by primer pair of oligo-dT and primer F-09.

### Sequence analysis and phylogenetic comparisons

The nucleic acid sequences and deduced amino acid products were analyzed and assembled using the DNASTAR program (Lasergene). BLAST searches were carried out using the NCBI server (www.ncbi.nlm.nih.gov) with all available databases. An ORF search was performed with the ORF Finder of the NCBI. Conserved domains in the amino acid sequences were identified using the CD-Search of the NCBI [28]. The secondary structures of the 5′-UTR and 3′-UTR were modeled with the Mfold program [29]. Previously determined sequences of members of family *Tymoviridae* were downloaded from GenBank and used to build the phylogenetic trees. Sequences were aligned using the CLUSTAL_X, version 2.0 software [30]. Calculations of nucleic acid and amino acid sequence identities were performed using the MEGA version 4 software [31]. Neighbor-joining phylogenetic trees of RP and CP were also generated in MEGA, using the p-distance and the Poisson correction algorithms. The robustness of the branching was evaluated by bootstrapping using 1,000 replications.
